# Development of Chemometric Models Based on a LC-qToF-MS Approach to Verify the Geographic Origin of Virgin Olive Oil

**DOI:** 10.3390/foods10020479

**Published:** 2021-02-23

**Authors:** Ina Willenberg, Alessandra Parma, Anja Bonte, Bertrand Matthäus

**Affiliations:** Working Group for Lipid Research, Department of Safety and Quality of Cereals, Max Rubner-Institut (MRI), 32756 Detmold, Germany; alessandra.parma27@gmail.com (A.P.); anja.bonte@mri.bund.de (A.B.); Bertrand.Matthaeus@mri.bund.de (B.M.)

**Keywords:** olive oil, authenticity, geographical origin, mass spectrometry, logit regression

## Abstract

In the presented study a non-targeted approach using high-performance liquid chromatography coupled to electrospray ionization quadrupole time-of-flight mass spectrometry (HPLC-ESI-qToF-MS) combined with chemometric techniques was used to build a statistical model to verify the geographic origin of virgin olive oils. The sample preparation by means of liquid/liquid extraction of polar compounds was optimized regarding the number of multiple extractions, application of ultrasonic treatment and temperature during concentration of the analytes. The presented workflow for data processing aimed to identify the most predictive features and was applied to a set of 95 olive oils from Spain, Italy, Portugal and Greece. Different strategies for data reduction and multivariate analysis were compared. Stepwise variable selection showed for both applied multivariate models—linear discriminant analysis (LDA) and logit regression (LR)—to be the most suitable variable selection strategy. The 10-fold cross validation of the LDA showed a classification rate of 83.1% for the test set. For the LR models the prediction accuracy of the test set was even higher with values of 90.4% (Portugal), 86.2% (Italy), 93.8% (Greece) and 88.3% (Spain). Moreover, the reduction of features allows an easier following up strategy for identification of the unknowns and defining marker substances.

## 1. Introduction

Food fraud is not a new phenomenon [1]. Since food is traded, counterfeiters can come up with ways to manipulate food in such a way that buyers do not find a diminished value. In earlier times they used simple falsifications, such as addition of sand to flour or water to wine or oil [2]. Nowadays, the possibilities for falsification are much more sophisticated and also the starting points for falsification are more diverse. While in the past it was a matter of stretching the food with inferior materials, today buyers are also deceived about other quality features such as wrongly declared geographic origin of the food or illicit processing [3].

The EU has recognized this problem when it adopted the new regulation (EU) 2017/625, on 15 March 2017 for a more effective fight against food fraud [4]. For the first time there was a paradigm shift from the almost exclusively risk-based approach to food safety of Regulation (EU) 882/2004 considering and avoiding a risk of food adulteration. As a consequence, the National Reference Centre for Authentic Food (NRZ-Authent) in Germany was established in 2017 [2,3]. Moreover, the search for reliable and robust methods for the detection of food fraud is an ongoing story and many research groups dedicate a lot of time to this topic [5,6,7,8,9].

One of the foods most affected by adulteration is olive oil; it is among the top 10 of the most adulterated food, although it is the best regulated and protected edible oil by several EU regulations (e.g., Regulation (EU) No 1308/2013, Regulation (EEG) No 2568/91 or Regulation (EU) No 29/2012)) or specifications of the International Olive Council (IOC) [10]. These regulations and specifications are supposed to help classifying the different olive oil categories but questions regarding authenticity of the geographic origin of the oil given on the label or processing are hardly to be answered with these methods.

Apart from quality parameters such as sensory perception or price also the geographical origin of the product determines the buying decision of consumers. White and Cundiff already showed in 1978 the country of origin to be a salient cue in buyers’ perceptions of quality [11]. Later Al-Sulaiti and Khalid stated that the country of origin may be an important element in the perceptions consumers have of products and services especially where little other information is known [12]. For extra virgin olive oil Caporale et al. found that its typicality is strongly affected by the origin of the raw material and the manufacturing technology [13]. In addition, the geographical origin of olive oil strongly influences the prices for the oils. For example, extra virgin olive oil from Italy is usually much more expensive than olive oil from other southern European countries [14] resulting in a great potential for profit maximization by incorrect labelling.

In order to take account of the consumers’ need for information on the geographic origin of olive oil, Regulation (EU) 29/2012 defined in Article 4 that indications of origin are compulsory on the labelling, but only for extra virgin and virgin olive oil [15]. However, the official methods given in Regulation (EEG) No 2568/1991 do not allow verifying the geographic origin of olive oil. Recently, several papers on the differentiation of olive oil from different regions using different analytical methods combined with multivariate techniques have been published. Such methods are MS-based techniques [16,17], HPLC [18], genomics [19], stable isotope ratio analysis [5,20], NMR [9,21,22], NIR [7] or two-dimensional GC-MS [23]. Regarding the application of NMR in questions of origin verification there are only few studies available, mostly based on 1H-NMR as the generation of quantitative data is faster and simpler in comparison to 13C-NMR [21]. The common sample preparation for NMR analysis comprises simple dissolution of the oil in deuterated organic solvent, which results in spectra dominated by the fatty acids. In order to respond to minor components of the oil pulse experiments were developed [22]. In a recent study an alternative sample preparation was suggested, which allows to focus on the minor components like phenols, aldehydes and ketones without multiple pulse sequencing [9]. The presented model based on the developed extraction protocol allowed to differentiate olive oils from Italy, Greece and Spain with a correct classification of 96% during cross-validation [9]. Another analytical technique used for origin determination of olive oils is NIR [7,24]. The advantages of NIR spectroscopy are that no sample preparation is needed and the time for analysis per sample is short which makes it feasible to analyze a huge set of samples as needed for the development of reliable chemometric models. Moreover, once the calibration for typical identity and quality parameters, like fatty acid and triacylglycerol composition, free fatty acids or peroxide value is established a multitude of parameters can be determined within one single analysis. As a result, the chemometric model build for origin verification can not only be based on the spectra but also on the classical parameters determined by NIR, as suggested by Gertz et al. [7] who presented an approach based on the analysis of more than 5000 olive oil samples and different statistical approaches to identify the origin of olive oils by NIR.

In addition to these spectroscopy-based methods different MS techniques were also suggested for the authentication analysis of olive oils. Although MS-based methods do not play a role for the legal control of identity and quality of olive oils as defined in Regulation (EEC) No 2568/1991 or in the methods standardized by the International Olive Council, MS-technologies become more and more important in the field of authenticity control.

Such questions are often addressed by non-targeted approaches. After data processing of a set of samples with known characteristics (e.g., geographic origin) the derived features are used for the development of statistical models allowing to predict the relevant characteristic for an unknown sample. In order to detect as much compounds as possible, the analysis is often carried out using different ionization polarities or even different chromatography systems (liquid chromatography (LC) and gas chromatography (GC)) within one comprehensive approach [16,17]. An advantage of the usage of MS instruments with high mass resolution and accuracy is the possibility to identify the most predictive features based on the MS and MS/MS data in order to gain more substantial information related to the distinct question which can additionally help developing other approaches, e.g., targeted ones [16].

Especially the use of GC-MS methods analyzing volatile compounds of olive oil to support the sensory assessment of panel tests was one of the first applications for MS methods measuring a huge number of unknown compounds [25,26]. Nowadays it becomes more and more popular and according to Aparicio-Ruiz et al. this approach explaining virgin olive oil aroma descriptors is fully accepted and demanded by the olive oil sector [27]. Later other parameters such as triacylglycerols and fatty acids [28] or sesquiterpene hydrocarbons [29] measured by different GC-MS techniques were used in combination of chemometric methods for the discrimination of samples from different origin.

In the field of olive oil authentication only few LC-qToF based approaches were reported, e.g., addressing the determination of the quality category (extra virgin, virgin, lampante) by GC-APCI-qToF [30] or differentiating between varieties by means of LC-ESI-ToF and GC-APCI-ToF [31] or LC-ESI-QTOF-MS [8]. Regarding origin verification Gil-Solsona et al. presented an LC-ESI-qToF-MS approach based on 90 oils from different Spanish regions which enables to identify chemical markers for the differentiation of the regions [16]. Recently, Olmo-Garcia et al. applied the combination of two platforms (LC-ESI-qToF-MS and GC-APCI-qToF-MS) in order to discriminate and identify possible origin markers for six regions in Spain, Greece, Italy and Morocco [17]. It is worth to mention that the number of factors influencing the discrimination of different regions is lower in comparison to countries. Olive oils from different regions are often from the same variety, growing under similar climate conditions and harvested and processed by similar devices resulting in a narrower variation while the variation over the whole country is much more diverse. This often allows the discrimination of olive oils from different regions whereas the separation of oils from different countries is difficult since the huge variation leads to overlapping between samples from several countries. In a preliminary study with data on the fatty acid profile Winkelmann and Küchler showed a satisfactory differentiation between oils from two distinct regions, but much more overlapping when data from all regions in two countries were considered [9].

The present work aims to present the workflow for differentiating olive oils from different countries of origin (Spain (Esp), Italy (Ita), Portugal (Pt), Greece (Gre)) by using a non-targeted HPLC-ESI-qToF-MS approach based on the polar extract of olive oils. In contrast to the work of e.g., Olmo-Garcia et al. [17] the current approach should not only enable the differentiation of particular regions from different countries but includes samples from the whole area of the investigated countries. The development and optimization of the analytical method is demonstrated and discussed and the data reduction by statistical methods is described. One major focus is the application of logit regression (LR) for the verification of origin. Up to now this promising binary approach is rarely used in the evaluation of origin authentication in olive oil. For this purpose, a set of 95 samples with known origin was analyzed and linear discriminant analysis (LDA) and logit regression (LR) was applied for differentiating between geographic origins.

## 2. Materials and Methods

### 2.1. Chemicals

Hydroxytyrosol, tyrosol, vanillin, vanillic acid, ferulic acid, p-coumaric acid, cinnamic acid, luteolin and apigenin were used as reference standards for the optimization of the extraction procedure and purchased from Sigma-Aldrich (Schnelldorf, Germany). Methanol in HPLC-grade quality was used for the extraction (Baker, Avantor Performance Materials, Arnhem, Netherlands). For HPLC-qTof-MS analysis formic acid (Honeywell Fluka, Fisher Scientific, Schwerte, Germany), methanol (Fisher Scientific, Schwerte, Germany), isopropanol (99.9%, Honeywell, Riedel-de-Häen, Fisher Scientific, Schwerte, Germany), 1M sodium hydroxide solution (Agilent Technologies, Santa Clara, CA, USA) and water (Merck, Darmstadt, Germany) were used.

### 2.2. Samples

Virgin olive oil samples from Italy (*n* = 39, e.g., from Lazio, Calabria, Sicily, Sardinia, Apulia, Basilicata, Tuscany, Abruzzo), Spain (*n* = 26, e.g., Andalucia, Castilla La Mancha, Catalonia, Extremadura), Greece (*n* = 13, e.g., from Aegean Islands, Peloponissos) and Portugal (*n* = 17, e.g., from Tras-os-Montes, Alentejo) were submitted from different producers to a Swiss olive oil panel with the aim to participate in an olive oil competition. The oils were derived from different varieties and the set of samples included monovarietal oils as well as blends from different varieties. All of the related information to the sample, e.g., the geographic origin, were provided directly by the producer or distributor of the oil.

### 2.3. Sample Preparation

Liquid/liquid extraction (LLE) with methanol/H2O (80/20 (*v*/*v*)) was applied to prepare the polar extract of the oils which was subsequently analyzed by HPLC-qToF-MS. LLE procedure was optimized regarding the following parameters: number of multiple extractions (one up to three extraction steps, the different extraction steps were not combined but analyzed independently from each other), the application of ultrasonic treatment (15 min, yes or no), and the temperature during the evaporation of the extraction solvent (room temperature, 30 °C, 40 °C). The optimized and finally applied sample preparation by LLE procedure was carried out as follows: approximately 1.5 g of olive oil was exactly weighed in a 10 mL reaction tube. After addition of 1.5 mL extraction solvent (methanol/H2O 80/20 (*v*/*v*)) the 2-phase system was shaken vigorously for 1 min at 1500 1/min (VXR basic Vibrax, IKA-Werke, Staufen, Germany). Next, the extraction solution was centrifuged (3000 rpm, 5 min) and 1 mL of the resulting supernatant was collected into a new 10 mL glass tube. The remaining lower phase was extracted a second time in the same manner and 1.5 mL of the second supernatant was combined with the first extract. The combined extract was evaporated to dryness at 30 °C with a stream of nitrogen for 75 min (Reacti-Therm, Thermo Scientific, Schwerte, Germany). The residue was dissolved in 0.75 mL of methanol/H2O 50/50 (*v*/*v*), shaken vigorously for 1 min (1500 1/min) and transferred to a 1.5 mL reaction tube. After centrifugation for 5 min at 5000 rpm the supernatant was transferred into a HPLC vial with glass insert. Samples were stored in the dark at 4 °C till analysis.

Inter-day repeatability of the optimized extraction procedure and the MS performance was tested by analyzing the same olive oil sample (*n* = 5) at three different days within five days. For this purpose, the areas of the signals of OH-tyrosol, vanillic acid, vanillin, p-coumaric acid, ferulic acid, cinnamic acid, luteolin and apigenin from its extracted ion chromatograms were evaluated.

### 2.4. HPLC-ESI-qToF-MS Analysis

The untargeted analysis of the polar extract was carried out by HPLC-ESI-qToF-MS. For HPLC (Dionex Ultimate 3000 Series, Thermo Fisher Scientific, Waltham, MA, USA) samples were kept in the autosampler at 5 °C until injection of 10 µL. Separation was carried out at 40 °C on a 1.7 µm, 150 mm × 2.1 mm EVO C18-reversed phase Kinetex column with a SecurityGuard ULTRA sub-2 µm pre-column (Phenomenex, Torrance, CA, USA). An aqueous solution of 0.01% formic acid was used as eluent A, and methanol acidified with 0.01% formic acid was used as eluent B. The following gradient was used: 0.00–5.00 min linear 10–30% B, 5.00–14.00 min linear 30–60% B, 14.00–14.50 min linear 60–70% B, 14.50–14.51 min 70–100% B, 14.51–16.00 min isocratic 100% B, 16.00–16.01 min return to initial conditions of 10% B, 16.01–18.00 min isocratic 10% B 15. The detection was carried out after negative electrospray ionization (ESI) by a qToF-MS (Maxis Impact HD, Bruker Daltonik, Bremen, Germany) operating in broadband collision induced dissociation (bbCID) mode. Ionization was carried out in negative mode as it can be assumed that the analyzed extract contains rather polar compounds, e.g., phenolic compounds due to the sample preparation strategy. Several studies described that analysis of this kind of compounds did get sufficient or better results in negative mode compared to positive mode in most cases [32,33,34]. Nitrogen was used as desolvation and nebulizing gas. MS data were acquired over a *m*/*z* range of 50–1000 using the following parameters: capillary voltage 3000 V, drying gas temperature 200 °C, dry gas flow 8 L/min, nebulizing gas pressure 2 bar and plate offset −500 V. Compass otofSeries 1.7 package (Bruker Daltonic, Bremen, Germany) was used for data acquisition. For the first 30 s of every run a calibration solution of 10 mM sodium formate (12.5 mL H2O, 12.5 mL isopropanol, 50 µL formic acid, 250 mL 1M NaOH) was infused to the MS with a flow of 0.18 µL/min. Samples were analyzed randomly and in duplicate. Every ten samples a blank sample (methanol/H2O 50/50 (*v*/*v*)) was injected. In order to check performance of the extraction and the MS instrument one quality control (virgin olive oil) was extracted per day together with the samples used for building the chemometric model. The quality control was repeatedly injected every 30–40 samples. The evaluation of the reproducibility for the quality control was carried out as described for the optimization of the extraction process. A total ion chromatogram of an exemplary olive oil sample can be found in Figure S1.

### 2.5. Data Processing

The following data processing was performed with all olive oil samples and the blank samples. Internal calibration and peak detection were performed using Data Analysis 4.2 (Bruker, Bremen, Germany). First step of data processing comprised the internal recalibration of every sample using sodium formate in negative mode as calibrant. Next, peak detection was carried out for every sample using the Find Molecular Features Algorithm. The following settings were used for compound detection: S/N threshold 5, correlation coefficient threshold 0.9, minimum compound length 7, smoothing width 4, negative adducts used for feature detection: M-H, M-H2O-H, M+Na-2H, M+K-2H, M+Cl, M+HCOOH-H, 2M-H, 2M+HCOOH-H. Every detected feature was characterized by retention time and *m*/*z* ratio. The combination of the independently detected features of all samples of the data set (95 olive oils) within one bucket table was carried out using Profile Analysis 2.1 (Bruker, Bremen, Germany). Advanced bucket table generation was performed within the retention time range from 0.5 min to 17.0 min and a mass range from *m*/*z* of 50 to 1000 using a bucket width of 0.5 s and 5 mDa. The mass of the most intense isotope was used for bucketing

For further data analysis the olive oil samples were grouped according to their geographical origin (Italy, Greece, Spain, Portugal) and all blank samples were assigned to one group. In the next step, the MS data were processed according to the following criteria: in order to keep a distinct bucket (i) the mean of at least one group of origin has to be two-fold higher as the mean of the blank group, (ii) the value of the bucket has to be higher than zero for more than 50% of the samples of at least one group, (iii) at least two-fold change of the mean from one group to another group. Between step (ii) and (iii) the mean of the duplicates was calculated. The reduction of buckets was performed using Excel 2010 (Microsoft, Redmont, WA, USA).

### 2.6. Chemometric Analysis

Different strategies to optimize the number and selection of variables, which are used for building the classification model by LDA or LR were applied. In one approach principal component analysis (PCA) was applied to reduce the number of variables and dimensionality. The number of principal components (PC) used for LDA was optimized based on the classification error of a 10-fold cross validation. An alternative approach compared the differences of the means per group by ANOVA followed by Tukey post-test. Variables were maintained if at least two group means differ significantly within this test (*p* < 0.05). In a next step, the Pearson correlation coefficients of the features were calculated and features at the same retention time were removed when the correlation coefficient was below −0.8 or above 0.8. The last step of feature reduction was based on stepwise variable selection. Variable selection by stepwise LDA was performed using forward selection. The number of variables was optimized based on highest validation entropy Rsquare value and lowest validation misclassification rate for one test set. LR variable selection was carried out using forward selection and Bayesian information criterium as stopping rule. Variable selection for LR models was carried out individually for every LR model (Ita vs. others; Gre vs. others; Esp vs. others; Pt vs. others) with all 95 samples in the training set. The data sets obtained by the different strategies were used for building classification models depending on the geographic origin based on LDA and LR. As LR is a binary regression approach, one model was calculated for verifying the geographic origin of each country (e.g., Ita vs. non-Ita). 10-fold cross validation was applied to evaluate the predictive abilities of the different LDA and LR models. Each sample was randomly assigned to a test set once, resulting in ten test sets, each with nine or ten samples. As due to the availability of authentic sample material the number of samples per country is not ideally balanced between the considered countries, the number of samples per country was predefined for each test set (ES: 2–3; Gre: 1–2; Ita: 3–4; Pt: 1–2). The different approaches for feature reduction and the application of LDA and LR were compared. Chemometric analysis (LDA, LR, stepwise variable selection) was conducted using JMP 14.3.0 (SAS Institute, Cary, NC, USA).

## 3. Results and Discussion

### 3.1. Optimization of the Sample Preparation

Even when applying non-targeted approaches, a reliable and robust sample preparation plays an important role and has a great influence on the results. In comparison to the analysis of the non-polar extract by simple dilution of the oil sample with organic solvent, the use of the polar extract offers the advantage that even minor components, e.g., phenols are concentrated and consequently recorded in the non-targeted approach. Thus, sample preparation mainly determines the classes of compounds on which the subsequent classification model is based. For preparing the polar extract of edible oils mainly solid phase extraction (SPE) with diol or C18 cartridges [32,35] or LLE with methanol/H2O as extraction solvent is used [35]. However, Mateos et al. obtained no significantly different results when applying LLE or SPE [35]. Moreover, for a non-targeted approach not aiming to absolutely quantify single compounds it is rather important to choose a sample preparation which is easy to handle and robust as well as reproducible than to focus on a complete extraction. Additionally, for the development of chemometric models it is necessary to analyze a large number of samples and therefore a fast and less laborious sample preparation is useful. For these reasons LLE was applied in the present study. In comparison to SPE protocols no disposable SPE cartridges were needed and the use of organic solvents was diminished as only 3 mL of extraction solvent was needed per sample. SPE protocols often have a higher consumption of solvents as the cartridges usually have to be cleaned and conditioned before loading the sample and the following washing and eluting steps require the use of solvents, too.

**Figure 1:**
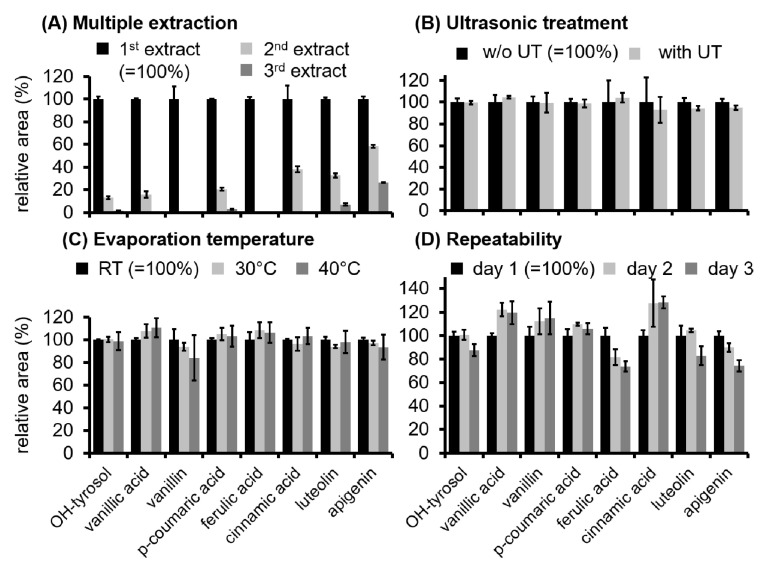
Effect of multiple extraction (analyzing three sequential extraction steps of the same sample separately, (**A**), ultrasonic treatment (**B**) and evaporation temperature (**C**) on extraction efficiency. For the repeatability (**D**) the same oil was extracted and analyzed independently on three days. Shown are mean ± SD (*n* = 3). Results of statistical tests related to part (**B**–**D**) of the figure can be found in the additional information S1 in the Supplementary Materials.

Before analyzing the set of olive oils from different geographic origins the LLE procedure was optimized regarding the extraction efficiency and optimal handling and characterized according to the repeatability. In order to determine the required number of multiple extraction steps, three sequential extracts were analyzed separately and the area of exemplary analytes belonging to different phenolic classes (OH-tyrosol, vanillic acid, vanillin, p-coumaric acid, ferulic acid, cinnamic acid, luteolin and apigenin) were evaluated. Figure 1A shows the relative areas of the second and third extraction step in comparison to the first extract. The second extract still contained significant amounts of cinnamic acid (38%), luteolin (33%) and apigenin (53%) in comparison to the first extract. In comparison to the first extract, in the third extract the percentage for most of the compounds was found to be below 10%, only apigenin showed a higher percentage of still 26%. The different behavior of the phenols may be explained by the polarity of the compounds: the used extraction solution consisting of methanol/H2O 80/20 (*v*/*v*) seems to be almost ideally suited for the extraction of the more polar compounds like OH-tyrosol, vanillic acid and p-coumaric acid. Nevertheless, with decreasing polarity of the considered compounds, the percentage of these compounds in the second and third extract is increasing (e.g., for luteolin and apigenin), probably because of a lower extraction efficiency of the used solvent for these less polar compounds. For non-targeted approaches the extraction process ideally has to be suitable for the extraction of compounds with a wider polarity range. The choice of the extraction solution and the number of multiple extractions is always a compromise and cannot focus on the complete extraction of every compound. Moreover, as already explained above the complete extraction of all compounds is not the primary goal for a sample preparation applied for a non-targeted approach. The most important is to achieve a reproducible extraction yield for the analytes. According to these considerations a two-fold extraction process was applied in the further work of the presented study as it reduced the effort for sample preparation in comparison to a third extraction step without decreasing extraction efficiency dramatically.

In a next step the effect of ultrasonic treatment on extraction efficiency was investigated. This treatment is often applied in LLE protocols for the extraction of phenols for improving the efficiency of the extraction [36]. As shown in Figure 1B ultrasonic treatment did not enhance the extraction of the considered phenols in the current protocol. Therefore, ultrasonic treatment was not applied in the subsequent experiments.

A further parameter which was optimized addressed the handling of the evaporation of the extraction solvent in order to concentrate the extracted analytes. Evaporation of the solvent was carried out using a stream of nitrogen to avoid oxidation of susceptible phenolic compounds resulting in uncontrollable modification of the analytes. The time needed for the evaporation to dryness might be shortened by the application of heat. Since phenolic compounds are thermally labile and may degrade during the evaporation process at higher temperature, the effect of heat treatment during evaporation has to be carefully checked. For this purpose, several extracts derived from the same olive oil were evaporated at room temperature, 30 °C and 40 °C. As to be expected, the time needed for evaporation to dryness decreased with increasing temperature. Figure 1C demonstrates that application of 30 °C and 40 °C did not result in significant losses of the exemplary phenols in comparison to the evaporation process carried out at room temperature. Evaporation at 30 °C decreased the time needed for evaporation by more than 60 min compared to room temperature without affecting the concentration of phenols. As a further increase of evaporation temperature to 40 °C did not dramatically shorten the evaporation time, a temperature of 30 °C was defined for the final protocol.

**Figure 2:**
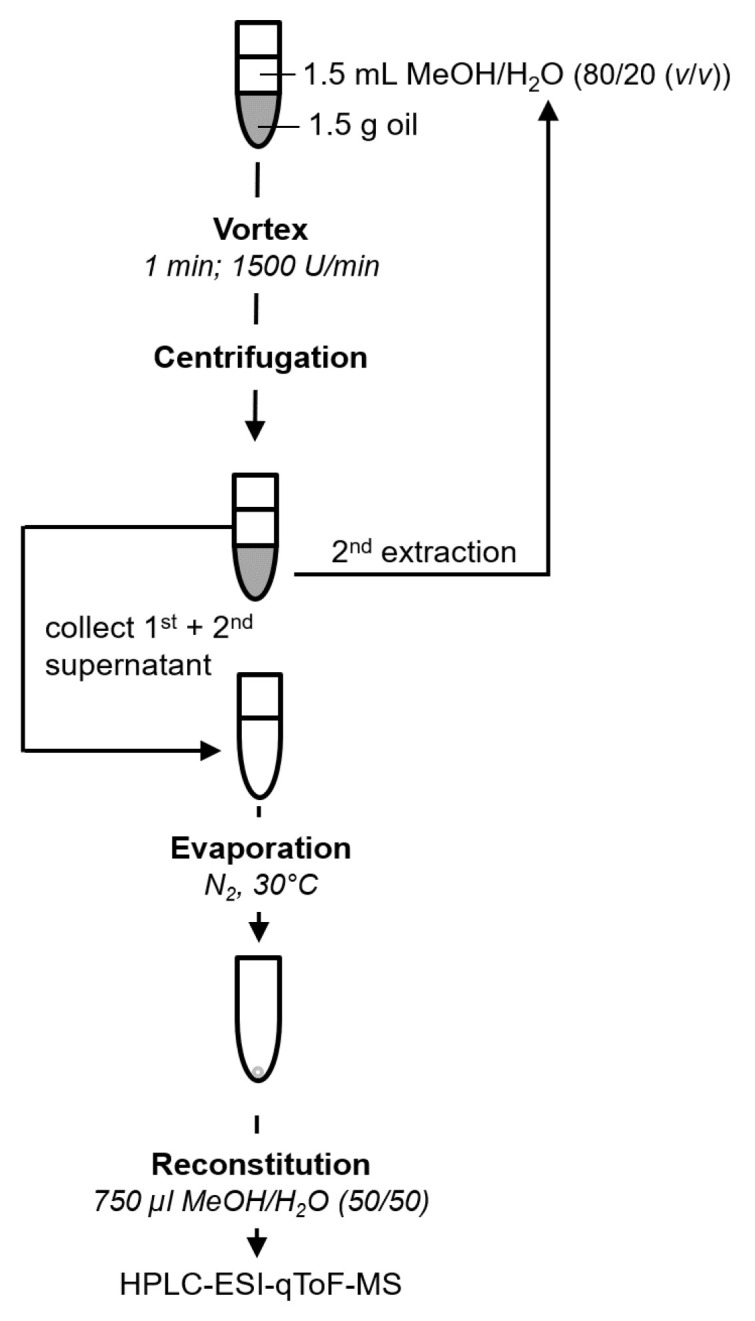
Optimized procedure for sample preparation.

Based on these preliminary tests addressing an effective but preferable easy and less laborious sample preparation the final LLE procedure was defined (Figure 2). The protocol comprised a simple two-fold extraction with a solution of methanol/H2O 80/20 (*v*/*v*) without application of ultrasonic treatment. Evaporation to dryness to concentrate the analytes was performed using a stream of nitrogen at 30 °C. Application of the final protocol to the same virgin olive oil at three independent days showed acceptable results for inter-day repeatability (Figure 1D). This result demonstrated that the proposed protocol is ideally suited for the usage in non-targeted profiling studies as it delivers reproducible results in an acceptable preparation time which is a key fact for this kind of application. The suitability of the extraction and the MS performance was supported by the evaluation of the quality control, which was extracted once per day in parallel with the samples of different origin. For the exemplary phenols which were evaluated in all of the analyzed quality control samples the relative standard deviation of the corresponding area was not higher than 10.2% (OH-tyrosol 6.0%, vanillic acid 9.0%, p-coumaric acid 6.0%, ferulic acid 9.0%, luteolin 10.2%, apigenin 6.0%) indicating a good reproducibility of extraction and analysis which allows to further evaluate and compare the data set of the samples from different origin.

### 3.2. Data Processing

In addition to sample preparation data processing is another key step for the calculation of a prediction model for the differentiation of the country of origin of extra virgin olive oils based on analytical data. The aim of such a data processing must be to reduce the huge data volume to the information that is necessary for the differentiation of the samples. In an ideal case only some few features remain after the successful data processing. Here it has to be kept in mind that the most important compounds with significant differences between the different groups of samples not necessarily have to be identified. For the development of a prediction model it is sufficient that the compounds are unambiguously assigned by data processing and named with unique names.

In our untargeted approach peak detection and bucket table generation was performed automatically with the Profile Analysis Software. Peak detection using the Find Molecular Features Algorithm resulted in approximately 2000 features per sample. Based on the combination of ions derived from the same compound, e.g., isotopes, charge states or adducts this peak finding algorithm creates so-called features which correspond to a distinct compound. In a next step bucketing of all samples of the data set was carried out using the advanced bucketing tool. Combination of the features of the 95 samples derived from Greece, Italy, Spain and Portugal resulted in approximately 24,700 buckets. This rather high number of features was stepwise reduced by applying different criteria in order to identify the most predictive features and eliminate irrelevant variables caused by biological variability or experimental noise [37]. For this purpose, the different samples were assigned to four groups according to the geographic origin (Italy, Greece, Spain, Portugal) and different manners of comparison of the group mean values with the means of other groups or the blank samples were applied to reduce the number of features. Removal of all features which group mean was not at least for one group two-fold the value of the blank resulted in only 2.8% reduction of all features (24,024 features left). However, the next criterium which took the number of “filled buckets” (bucket value > 0) per feature into account was more effective. The elimination of all features which were only present in less than 50% of the samples for all groups reduced the number of features again by more than 91% (2123 features left). A threshold of 50% were used instead of 80% to cope with both the need to use only most common features on one hand and not to lose important markers for specific geographic areas on the other hand.

The next criterion took the differences between the mean of the different countries into account. Only the features, which showed at least a two-fold increase in the intensity between the country with the lowest value and the country with the highest value were kept [38]. In this way the features could be slightly reduced to a number of 1214.

### 3.3. Chemometric Analysis

**Figure 3:**
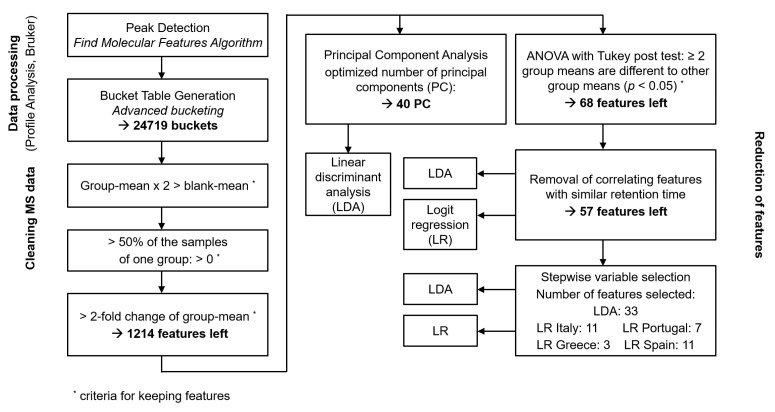
Flow chart of the process of MS data treatment including the different strategies for variable selection and application of the classification model.

Different strategies to identify or generate the most predictive variables from the resulting data set were tested in order to create a suitable basis for a classification model based on LDA or LR (Figure 3). In one approach with all of the remaining features after applying the two-fold criteria an ANOVA with Tukey post-test was performed and all groups which did not show a significant difference in means to at least two or more other groups (countries) were removed (*p* > 0.95). This approach resulted in a total number of 68 variables. In a next step, removal of correlating features was applied as the subsequently carried out LR is sensitive to highly correlating variables [39]. This removal of correlating features at the same retention time finally resulted in 57 features. It can be assumed that these 57 compounds described the differences between the four different groups most significantly and might be suitable variables for the classification models like LDA or LR. An alternative last step of feature reduction was carried out based on stepwise variable selection either using LDA or LR. Stepwise LDA resulted in 33 variables, while stepwise variable selection using LR identified 11 features for the model Ita vs. others, seven features for Pt vs. others, three features for Gre vs. others and 11 features for the model Esp vs. others. A list of the features which was used for the different models can be found in Table S1.

**Figure 4:**
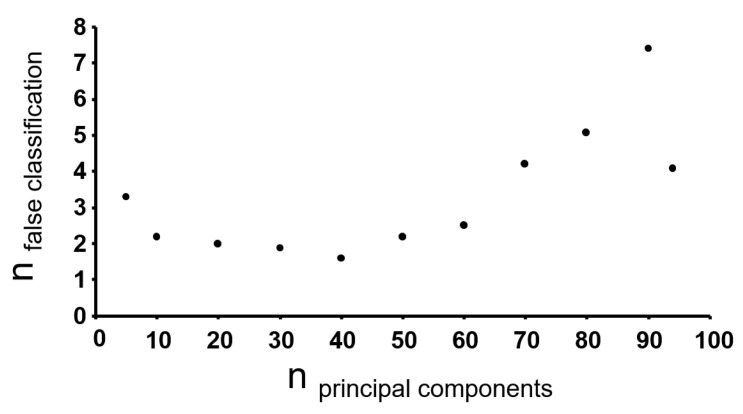
False classification rate of PCA-LDA as a function of different number of principle components used as variables for LDA.

As an alternative approach, dimensionality of the data after application of two-fold criteria was reduced by PCA. The optimum number of principal components (PC) was figured out by comparison of the classification error of the resulting LDA based on different numbers of PC. Figure 4 shows the number of false classified samples in the test set depending on the number of PC used for LDA based on a 10-fold cross validation. The classification error decreased with increasing number of PC up to a value of 40 PC, with more than 40 PC the classification error increased again, indicating an overfitting. Since the minimum classification error was obtained with 40 PC, this number of PC was used in the further course of this work. The summarized process of data processing and the different strategies for the reduction of features or dimensionality is shown in Figure 3.

In order to check the usefulness of the data reduction, LDA and LR was subsequently performed with the different sets of variables (Figure 3). The classification models based on LDA were built according to the geographic origin of the oils and the four countries Italy, Greece, Spain and Portugal were considered. Table 1 shows the results of a 10-fold cross-validation for the three models. The advantage of cross-validation is that the correctness of prediction is evaluated using a set of samples which is independent from the training set. Regarding the training set the overall correct classification rate was found to be 99.5% for the LDA based on the significant variables (ANOVA and Tukey post-test), 98.2% for the combination of PCA-LDA and 98.6% when applying stepwise variable selection (Table 1), indicating an excellent performance for all three variable selection criteria. For the more important independent test set the overall correct classification rate was observed to be 83.1% for the stepwise variable selection, 74.6% for the variable reduction by ANOVA and Tukey post-test and 83.3% for the combination of PCA-LDA (Table 1). As the evaluation of the prediction power based on the independent test set is more appropriate for unknown samples it has to be concluded that reduction of dimensionality by PCA or stepwise variable selection seemed to be the most suitable strategies with similar performance for the test set.

Although LDA is an often-applied approach for this kind of classification issues, there might be some limitations as this approach assumes the explanatory variables to be normally distributed and with equal covariances. Moreover, the observations per class should be balanced ideally [40]. For these reasons, LR was applied as an alternative classification model. The advantages of LR are that the predictor variables do not have to be normally distributed and that it is not necessary to have equal variances in the groups [39]. LR was carried out in a binary approach in order to verify the given geographic origin. For this purpose, it was necessary to conduct a separate model for each country (e.g., Italian samples vs. others including Spanish, Portuguese and Greek samples). As LR aims to predict the probability for a particular outcome (e.g., is the oil of Italian origin—yes/no) the resulting models will allow to verify the labelled origin of an unknown sample. For the verification of an Italian labelled sample, the model for the verification of Italian origin is applied and, similarly, the remaining models are used for samples with other declared origins. Table 2 summarize the overall correct classification rates for the different models using either variable selection based on significance differences (ANOVA with Tukey post-test) or stepwise variable selection. For the training sets correct classification rates above 96.5% were found for all models. The models based on variable selection with ANOVA resulted in correct classification rates for the independent test set of 72.4% (Pt), 75.8% (Ita), 88.3% (Gre) and 67.4%. As observed for the LDA approach, stepwise variable selection increased the predictive power for all models in comparison the variable selection solely based on significance (ANOVA with Tukey post-test) and resulted in correct classification rates of 90.4% (Pt), 86.2% (Ita), 93,8% (Gre) and 88.3% (Esp). 3D canonical score plots of the different LDA models can be found in Figure S2. In comparison to the LDA approach the correct classification rates of the different LR models are higher, indicating a higher predictive power for this approach which would make the LR the first choice for the studied question. One possible limitation using LR is that it can only be applied to verify a given (labelled) origin instead of classifying between the four countries. However, control authorities mainly focus on the verification of the labelling, which can be answered with the LR approach quite well. In addition to the higher predictive power of the LDA and LR models based on stepwise variable selection the further selection of variables might have also other advantages: An identification of the compounds which refer to the significant variables used for classification may open up the possibility of targeted analysis of these compounds. Verification of the origin based on a targeted approach might have advantages regarding the acceptance in official controls and can result in the need of less sophisticated analytical instruments, which availability may be limited. Due to the fact, that HRMS technology is an expensive way of analysis and secondly requiring a lot of user experience the next step has to be the identification of the potential marker compounds or compound compositions, as demonstrated by Olmo-García et al. to obtain a basis for developing a fast and easy to use targeted MS based method [17]. In this respect, HR-MS technology combined with chemometric models such as LR is an ideal combination to identify marker compounds which can be—after elucidation of the structure—used within targeted analysis to verify the geographic origin. The present study shows that it is feasible to identity suspicious samples on the basis of a few selected compounds in the polar extract of olive oil and that it is worth to identify these markers in a next step in order to generate prerequisites for a targeted method.

## 4. Conclusions

Within the first part of this work a suitable and reproducible protocol for the preparation of the polar extract of virgin olive oils which includes the phenolic compounds was developed for the application in an HPLC-ESI-qToF-MS method. As the approach was based on LLE the amount of solvents and consumables needed per sample were low, which was an ideal prerequisite to apply the protocol on a non-targeted metabolomics approach, for which lots of samples have to be analyzed in order to develop an appropriate model. Another important point is that the extraction method was fast and showed reliable results. In comparison to other LLE procedures [36,41] described, the amount of oil and solvent needed per sample is low, the ultrasonic treatment could be skipped as it does not enhance extraction efficiency. Moreover, due to the small volumes, the extraction can be performed in simple glass tubes which also increases the number of samples to be processed per time.

The second part of the present study demonstrated that the non-targeted screening of the polar fraction of virgin olive oils enabled classification of the country of origin by LDA or LR. As the sample set includes samples of only one crop year it is indispensable to verify this first, promising approach in multi-year analyses with a sufficiently large database. Although the study included oils of the main European producer countries, further studies should also take other countries into account and should address the question how blends of different countries behave within the models and suggest approaches how to deal with them. However, the study demonstrated that chemometric analysis, especially LR, can be an effective tool in detecting suspicious samples regarding the labelling of geographical origin of virgin olive oils. Although, such chemometric models cannot give a 100% prediction accuracy, a prediction accuracy of above 85% already helps strongly to increase the pressure on counterfeiters.

## Figures and Tables

**Table 1:** Classification rate of LDA based on different strategies of variable selection: PCA (40 principal components), ANOVA with Tukey post-test and removal of correlated features with similar retention time (57 variables) or stepwise variable selection (33 variables).

	Overall Correct Classification Rate (%) *	
Criteria Variable Selection	Training Set	Test Set
PCA	98.2	83.3
ANOVA with Tukey post-test/Removal of correlated features	99.5	74.6
Stepwise variable selection	98.6	83.1

* k-fold cross validation (k = 10, training set: 85 or 86 samples, independent test set: 10 or 9 samples).

**Table 2:** Classification rate of LR models based on different strategies of variable selection: ANOVA with Tukey post-test and removal of correlated features with similar retention time (57 variables) or stepwise variable selection (Ita: 11 variables; Pt: 7 variables, Gre: 4 variables; Esp: 11 variables).

	Overall Correct Classification Rate (%) *			
	Portugal	Italy	Greece	Spain
**ANOVA with Tukey post-test/Removal of correlated features**				
Training Set	100.0	100.0	100.0	100.0
Test Set	72.4	75.8	88.3	67.4
**Stepwise variable selection**				
Training Set	100.0	96.5	97.0	100.0
Test Set	90.4	86.2	93.8	88.3

* k-fold cross validation (k = 10, training set: 85 or 86 samples, independent test set: 10 or 9 samples).

## Data Availability

Data is contained within the article or supplementary material.

## Supplementary Materials

The following are available online at https://www.mdpi.com/2304-8158/10/2/479/s1, Figure S1: HPLC-ESI-qTOF-MS chromatogram (base peak chromatogram, MS1) of the polar extract derived from oilve oil, Figure S2: 3D Plot of LDA analysis, Table S1: List of features which was used for the different statistical models shown in Figure 3, Additional information S1: Results of statistical tests related to Figure 1.
