# Supplementary material for: Development of Chemometric Models Based on a LC-qToF-MS Approach to Verify the Geographic Origin of Virgin Olive Oil
Source: Foods. 2021 Feb 23;10(2):479. doi: 10.3390/foods10020479 (PMC7926913; doi:10.3390/foods10020479)
Supplement: Supplementary file 1 [file foods-10-00479-s001.zip › foods-1097784-supplementary.pdf]

Supplement Information

Development of chemometric models based on a LC-qToF-MS approach to verify the geographical origin of virgin olive oil

Ina Willenberg [1]*, Alessandra Parma[1], Anja Bonte[1], Bertrand Matthäus[1]

[1] Max Rubner-Institut (MRI), Department of Safety and Quality of Cereals, Working Group for Lipid Research, Detmold, Germany
* Correspondence: ina.willenberg@mri.bund.de

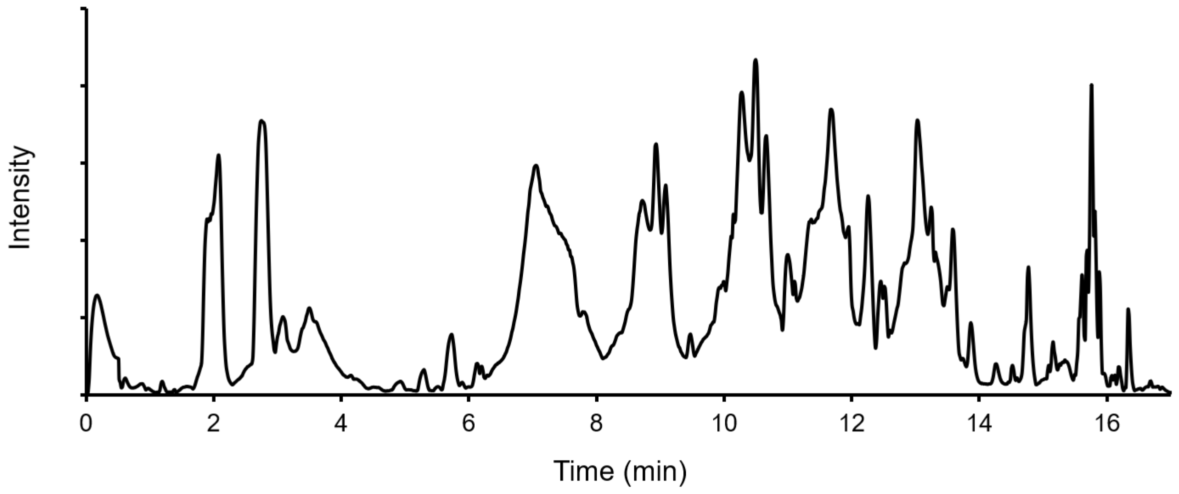

Fig. S1: HPLC-ESI-qTOF-MS chromatogram (base peak chromatogramm, MS[1]) of the polar extract derived from oilve oil.

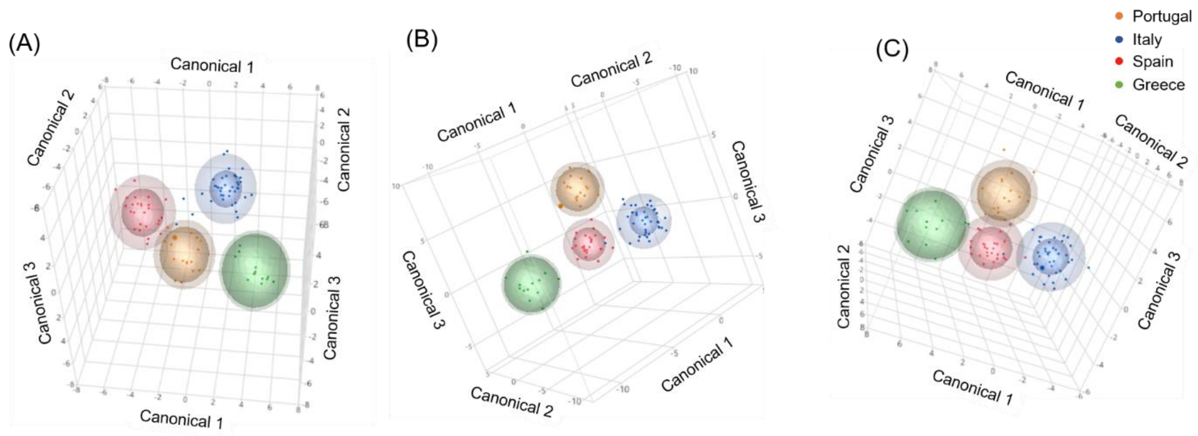

Fig. S2: Effect of number of variables used for linear discriminant analysis. Shown are normal 50% contours (outer circle) and mean CL elipses (inner circle) in the 3D contour plot for the LDA based on PCA-LDA (A), 57 (B) and 33 variables (C). The orientation of the 3D-Plot is optimized in order to show the most appropriate view.

Tab. S1: List of features which was used for the different statistical models shown in Fig. 3 of the manuscript.

	**57 features**		**stepwise variable selection**				
**Feature (retention time: m/z ratio)**	**LDA**	**LR**	**LDA**	**LR - Ita**	**LR - Pt**	**LR - Gre**	**LR - Es**
2.04min : 121.028m/z	x	x	x				x
2.57min : 151.076m/z	x	x	x				
3.00min : 509.161m/z	x	x					
4.83min : 136.016m/z	x	x	x	x		x	
5.84min : 151.039m/z	x	x	x			x	
5.88min : 225.040m/z	x	x					x
6.26min : 347.036m/z	x	x					
6.27min : 229.071m/z	x	x					
6.30min : 521.126m/z	x	x	x		x		
6.48min : 123.044m/z	x	x					
7.04min : 149.023m/z	x	x					
7.13min : 633.178m/z	x	x	x	x			
7.20min : 805.208m/z	x	x					
7.72min : 257.066m/z	x	x	x				x
7.85min : 635.193m/z	x	x	x				
7.88min : 425.143m/z	x	x					
8.34min : 609.215m/z	x	x	x				
8.61min : 395.132m/z	x	x	x				
8.99min : 391.102m/z	x	x	x				
9.44min : 407.097m/z	x	x					
9.44min : 409.112m/z	x	x	x				
9.51min : 152.034m/z	x	x		x	x		
9.70min : 391.102m/z	x	x	x	x			
9.74min : 719.224m/z	x	x	x				x
10.45min : 301.106m/z	x	x	x	x			
10.56min : 255.086m/z	x	x	x				
10.67min : 183.065m/z	x	x	x				
10.75min : 323.073m/z	x	x	x				
12.16min : 593.183m/z	x	x	x		x		
12.50min : 651.219m/z	x	x					
14.09min : 465.138m/z	x	x					
14.11min : 631.198m/z	x	x	x	x			
14.15min : 321.169m/z	x	x	x				x
14.43min : 331.117m/z	x	x	x				
14.69min : 317.138m/z	x	x			x		
14.72min : 431.128m/z	x	x					
14.77min : 347.148m/z	x	x		x			
14.96min : 409.185m/z	x	x					
15.00min : 663.187m/z	x	x					
15.04min : 393.117m/z	x	x	x				x
15.15min : 303.123m/z	x	x					

15.44min : 259.191m/z	x	x	x	x			x
15.54min : 253.179m/z	x	x			x		
15.59min : 241.180m/z	x	x	x		x	x	x
15.60min : 311.222m/z	x	x					
15.60min : 483.329m/z	x	x	x				x
15.64min : 433.221m/z	x	x	x	x			
15.68min : 279.195m/z	x	x					
15.68min : 361.198m/z	x	x	x				
15.72min : 575.356m/z	x	x	x	x	x		
15.73min : 635.376m/z	x	x	x				x
15.75min : 637.386m/z	x	x					
15.76min : 591.459m/z	x	x	x				
15.77min : 363.214m/z	x	x					x
15.84min : 569.474m/z	x	x	x				
15.85min : 571.359m/z	x	x					
16.07min : 361.128m/z	x	x	x				

Additional Information 1: Results of statistical tests related to figure 1

The results given in Figure 1 (B)-(D) were additionally evaluated by applying statistical tests to check for significant differences between the different groups (JMP 14.3.0; SAS Institute, Cary, North Carolina, USA).

Fig. 1 (B) – Ultrasonic treatment: Student's t-test comparing both groups (with/wo ultrasonic treatment) revealed no statistical difference for all of the phenols ($p > 0.05$, $\alpha = 0.05$).

Fig. 1 (C) – Evaporation temperature: ANOVA followed Tukey post-test revelaed no statistical differences for the different temperatures applied during evaporation of the solvent ($p > 0.05$, $\alpha = 0.05$).

Fig. 1 (D) – Repeatability: ANOVA with Tukey post-test revealed differences between the days ($p < 0.05$, $\alpha = 0.05$) for the displayed phenols. However, no uniform trend can be observed for all phenols regarding the different days which would indicate for a systematic error (either for MS response/performance or the extraction). The variations were considered by analyzing all samples randomly in duplicate (on different days).
